# Treating COVID-19: Targeting the Host Response, Not the Virus

**DOI:** 10.3390/life13030712

**Published:** 2023-03-06

**Authors:** David S. Fedson

**Affiliations:** Independent Researcher, 57 Chemin du Lavoir, 01630 Sergy, France; davidsfedson@gmail.com; Tel.: +33-4-5099-1306

**Keywords:** host response treatment, COVID-19, generic drugs, statins, ACE inhibitors, angiotensin receptor blockers

## Abstract

In low- and middle-income countries (LMICs), inexpensive generic drugs like statins, ACE inhibitors, and ARBs, especially if used in combination, might be the only practical way to save the lives of patients with severe COVID-19. These drugs will already be available in all countries on the first pandemic day. Because they target the host response to infection instead of the virus, they could be used to save lives during any pandemic. Observational studies show that inpatient statin treatment reduces 28–30-day mortality but randomized controlled trials have failed to show this benefit. Combination treatment has been tested for antivirals and dexamethasone but, with the exception of one observational study in Belgium, not for inexpensive generic drugs. Future pandemic research must include testing combination generic drug treatments that could be used in LMICs.

## 1. Introduction

The COVID-19 pandemic has had a devastating impact on global health. As of January 2022, estimates of excess deaths exceeded 20 million worldwide [1]. The pandemic’s impact on social and economic life throughout the world has been enormous [2,3]. Remarkably, the one bright spot has been the rapid development of COVID vaccines [4]. They are estimated to have saved tens of millions of lives [5]. Despite a call for vaccine equity from the World Health Organization (WHO) [6], vaccine nationalism has been the dominant theme of global vaccination. Low-and middle-income countries (LMICs) have encountered great difficulty in obtaining supplies of COVID vaccines [7]. The same problems apply to treatments [8,9,10].

Most efforts to develop COVID-19 treatments have focused on antivirals [10,11]; for the most part, these are monoclonal antibody preparations. There have been appeals for a coordinated system for organizing and financing global pandemic research and development [12], but no one has suggested a practical way to create such a system and make it accountable. In the meantime, important public health issues (e.g., masking) have become deeply polarizing and have led to highly politicized debate [13].

For more than a decade I have argued that the only practical response to a global pandemic would be to target the host response to infection using inexpensive generic drugs [14,15,16,17,18,19,20,21,22,23,24,25,26,27,28]. People who live in any country with basic healthcare would already have these repurposed drugs on the first pandemic day. I recently wrote, “If we already knew that these drugs could save lives, they could be used in every country that is still affected by COVID-19” [25]. Host response treatment could be especially important for LMICs, which have experienced great difficulty in obtaining meaningful and affordable supplies of vaccines and antivirals.

This article will discuss several issues related to the use of generic drugs (including but not limited to statins, ACE inhibitors (ACEis), and angiotensin receptor blockers (ARBs)) to treat patients with COVID-19. It will focus on their ability to reduce 28–30-day mortality (not that reducing hospitalization rates and ICU admissions is unimportant). I reviewed the rationale for this idea in many articles written during periods when an influenza pandemic was anticipated (especially Refs. [15,17,19,22]). The principles outlined in these earlier articles apply equally well to COVID-19 and, in fact, to any pandemic, regardless of cause. They might also improve the host response to critical illness caused by any pathogen.

## 2. The COVID-19 Pandemic

**Figure 1:**
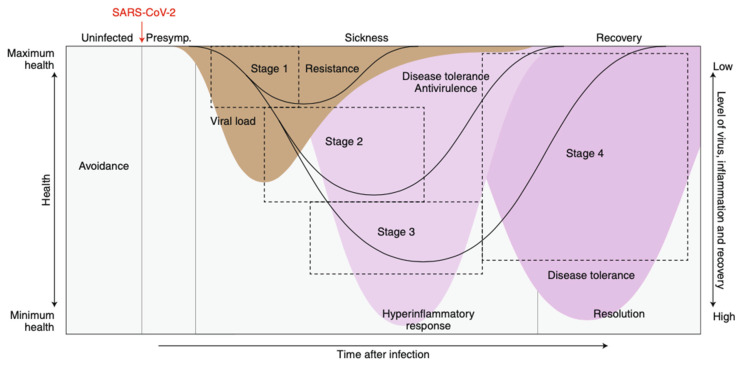
The relationship between disease stage and treatment for patients with COVID-19. Adapted from Ref. [66]. After SARS-CoV2 infection, the virus replicates and reaches peak levels during stage 1, after which levels steadily decline. As they decline, the host inflammatory response increases (the hyperinflammatory phase). This response eventually decreases and recovery begins. The relationships among these factors and the clinical course of the infection dictates which of the two defence strategies will be more effective. Before infection, avoidance is the best defence strategy. After infection, patients will be in a presymptomatic phase of the infection, which is followed by stage 1 with fever, malaise and other mild symptoms. Virus levels peak and then decline as patients exit stage I, independently of whether they will recover or progress to a severe or critical stage of infection. Antiviral drugs are more effective for asymptomatic individuals and patients in stage 1. By stages 2 and 3, the host inflammatory response drives the disease. If patients survive, the host inflammatory response subsides, resolution begins, and patients proceed toward recovery in stage 4. Drugs that treat the host response are more effective for patients in stage 4. Patients whose disease severity peaks in stages 1 or 2 can bypass later stages and enter directly into the recovery phase.

The pathophysiology of COVID-19 has been extensively reviewed [29,30,31,32,33,34,35,36,37,38,39,40], especially in relation to endothelial dysfunction [41,42,43,44,45,46,47,48,49,50,51,52,53,54]. The acute disease is also characterized by extensive modification of innate and adaptive immunity, increased inflammatory cytokines, abnormal interferon responses and immunothrombosis (Figure 1). This article will not review the pathophysiology of acute COVID-19 in any detail, but three points deserve emphasis. First, the many manifestations of acute COVID-19 (and probably other acute virus infections) reflect underlying differences in subphenotypes [55]. Host response treatment might affect only one of these many subphenotypes [56]. Second, the low mortality of COVID-19 in children and the much higher mortality in older adults must lie in their different evolutionary heritages [23,57,58,59]. The mechanisms underlying this difference remain to be determined. Third, findings for the pathophysiology and treatment of COVID-19 may apply to long COVID as well as the acute disease, even for those who initially had mild illness [60,61,62,63,64,65].

## 3. Randomized Controlled Trials vs. Observational Studies

Controversies over study methods—randomized controlled trials (RCTs) versus observational studies—continue to plague the literature on COVID-19. Most existing studies of treatments (antivirals and some of the drugs targeting the host response) are based on the results of RCTs, whereas much of the information on generic treatment is based on observational studies. The advantages and disadvantages of both study methods are summarized in Table 1.

Strong arguments have been made for the validity of observational studies for establishing the causal effects of treatments [67,68,69]. These reports and others [70,71,72,73] have criticized a sole reliance on RCTs for demonstrating treatment efficacy. Observational studies that use propensity scores have been shown to reliably mimic the results of RCTs [74]. Effectively managing the complex pathophysiology of COVID-19 probably requires using more than one drug; i.e., combination treatments are required [75]. A “pragmatic pluralism” is probably more suitable than a single method for establishing an effective approach to COVID-19 treatment [76].

## 4. Treatments for COVID-19

Treatments for COVID-19 can be divided into those that target the virus (antiviral agents) and those that target the host response to infection (often called immunomodulators). There may be some overlap between the two; drugs that modify the host response might also have antiviral effects. Comprehensive treatment guidelines have been issued for the U.S. by the National Institutes of Health [77] which offer extensive advice (448 pages) on treating non-hospitalized as well as hospitalized patients. For Europe, the European Respiratory Society (ERS) guidelines are much more succinct [78,79]. Treatment guidelines have also been issued by The World Health Organization (WHO) [80]. Table 2 summarizes both sets of guidelines. 

## 5. Treatments Targeting the Virus

Soon after the emergence of SARS-CoV-2, there was initial enthusiasm for treatment with either chloroquine or hydroxychloroquine (CQ/HCQ). Although in vitro evidence indicated these drugs might work, clinical trials and observational studies suggested they would be ineffective [81], a finding that recalled earlier clinical trials showing that CQ/HCQ did not work against other virus diseases (influenza and dengue) [82]. For remdesivir, clinical trials initially showed that intravenous treatment had no effect on 28-day mortality, although it appeared to shorten the length of hospital stay [83,84]. Two more recent RCTs [85,86] and an observational study [87] suggest that remdesivir treatment may actually reduce mortality by about 30–40% and prevent hospitalization but also may prolong the hospital length-of-stay. Studies of favipiravir have shown it offers no advantages over other ineffective antiviral agents [88,89]. The same lack of effect has been shown for ivermectin, an anti-schistosomal drug that has attracted a great deal of controversy [90,91,92,93]. In large studies, two other drugs that target the virus—colchicine [94,95] and convalescent plasma [96,97,98]—have also been shown not to reduce COVID-19 mortality. One hallmark of COVID 19 pathophysiology is disruption of normal interferon signaling [99,100]. Nonetheless, the WHO Solidarity trial [101] and studies of interferon-β [102] and pegylated interferon lambda [103] have shown interferon treatment does not reduce COVID-19 mortality.

Several monoclonal antibodies (mAbs) targeting SARS-CoV-2 have been developed [104] and tested to determine whether they reduce the severity of COVID-19 illness and its consequences [105]. A detailed Cochrane analysis of four RCTs included 9749 seropositive but unvaccinated, pre-omicron COVID-19 patients. It showed that pre-exposure prophylaxis with tixagevimab/ciligavimab probably reduced the number of symptoms and hospital admissions but had no effect on mortality [105]. A smaller study of casirivimab and imdevimab showed that treatment with these mAbs might have reduced symptomatic infections, but had uncertain effects on more severe symptoms and deaths. Two RCTs of post-exposure prophylaxis showed that the same two mAbs probably reduced the number of people infected but had little or no effect on mortality. A more recent study of Regdanvimab has shown a modest reduction in mortality [106]. After the Food and Drug Administration decided to deauthorize casirivimab and imdevimab (they were ineffective against omicron subvariants of SARS-CoV-2), there was a slow decline in their use [107]. Although most monoclonal antibodies are less effective against omicron subvariants, bebtelovimab appeared to be the most effective mAb against these subvariants [108] but it has since been withdrawn, because it is not effective. In addition, mAbs require parenteral administration, a feature that mostly limits their use to hospitalized patients.

Among antiviral agents shown to be effective against COVID-19 in unvaccinated adults, Paxlovid (nirmatrelvir/ritonavir) and molnupiravir have been shown to reduce symptomatic infections and hospital admissions [109,110]. During the recent omicron surge, Paxlovid reduced mortality in patients over 65 years of age, but not in younger individuals [111]. A small proportion of patients have tested positive for SARS-CoV-2 soon after completing a five-day course of Paxlovid treatment [112,113]. This “rebound” appears to be a general phenomenon and is not unique to Paxlovid [114].

Although antiviral drugs are sometimes useful against COVID-19 [115], they have several disadvantages. First, not all of these drugs are available as oral preparations; some (e.g., mAbs) require intravenous or subcutaneous administration, which may require hospital care. Second, supplies of many antiviral drugs are limited. The company that manufactures molnupiravir—an expensive antiviral—has negotiated agreements for supplying it to resource-poor countries at low prices [116]. However, molnupiravir is less attractive as an antiviral than Paxlovid because it appears to be less effective. Third, cost is still a barrier to widespread antiviral use, especially in resource-poor countries, because many of these drugs are still under patent.

As yet there are no descriptive data that document the global use of any antiviral agent for COVID-19 treatment. It is doubtful that any of these drugs have been or will be widely used in LMICs. Moreover, in patients who die of COVID-19, virus loads in the last days of life are far lower than they were when patients first tested positive (Figure 1) [117]. Because a dysregulated host response is largely responsible for disease severity at the end of life, treatments that target the host response to infection instead of the virus are more likely to improve patient survival.

## 6. Treatments Targeting the Host Response to Infection

Dexamethasone was the first drug shown to improve survival in hospitalized COVID-19 patients. In the RECOVERY RCT, dexamethasone reduced 28-day mortality in patients receiving mechanical ventilation (MV; rate ratio = 0.64) and in those requiring oxygen treatment without MV (rate ratio = 0.82), but not in those who received no respiratory support (rate ratio = 1.19) [118]. These results were not surprising: steroid treatment had been tested previously (with mixed results) in patients with sepsis and ARDS [119,120,121]. In. another RCT, intravenous dexamethasone decreased the need for MV but did not reduce 28-day all-cause mortality [122]. A WHO-sponsored meta-analysis of seven RCTs showed that dexamethasone and hydrocortisone decreased mortality (ORs = 0.64 and 0.69, respectively) [123].

Dexamethasone may work through its effects on endothelial dysfunction [124], but treatment is not without its hazards (e.g., hyperglycemia and opportunistic infections) [125]. Unfortunately, its use in non-hospitalized adults (for which there is no evidence of efficacy) has been considerable [126]. A recent observational study compared the results of steroid treatment with those from RCTs. The investigators argued that both methods could obtain similar results as long as the observational study methods were rigorous [127].

In spite of the encouraging results from RCTs, questions still remain about the role of steroid treatment in patients with COVID-19 [128]. Are currently recommended doses of dexamethasone immunomodulatory? Can responders and non-responders be identified before treatment starts? Who benefits most from steroid treatment? Do patients infected with only certain SARS-CoV-2 subphenotypes benefit from treatment? These and other questions will require ongoing attention from clinical and laboratory-based investigators.

A small retrospective cohort study showed that anakinra, an IL-1 receptor antagonist, reduced 21-day mortality but an RCT with 116 patients showed it failed to improve outcomes [129].

IL-6 is a prominent component of the “cytokine storm” seen in many seriously ill COVID-19 patients. Two RCTs have shown that tocilizumab (an mAb) reduces COVID-19 mortality [130,131]. In a WHO-sponsored meta-analysis [132], tocilizumab was associated with a reduction in 28-day all-cause mortality. The absolute mortality reduction (22%) was slightly less than that seen in patients who received dexamethasone instead of other corticosteroid preparations. In spite of the appearance of many subvariants of SARS-CoV-2, there have been no reports of tocilizumab’s reduced efficacy, probably because it targets IL-6, not the virus itself.

Fluvoxamine and fluvoxatine are selective serotonin uptake inhibitors (SSRIs) known to suppress cytokine levels and reduce COVID-19 mortality [133]. In a retrospective cohort study, both drugs reduced mortality (RR = 0.74; *p* = 0.04) [134]. Subsequent RCTs showed that fluvoxamine reduced hospitalization among outpatients [135,136], but did not reduce mortality [137].

Janus kinase inhibitors and tyrosine kinase inhibitors have also been tested for their effects on host response. In an early study, the Janus kinase inhibitor barcitinib was shown to dramatically reduce inflammatory cytokine levels and the need for oxygen therapy [138]. A later RCT showed that treating hospitalized adults with COVID-19 pneumonia with another Janus kinase inhibitor (tofacitinib) reduced 28-day and 60-day mortality and 28-day all-cause mortality (HR = 0.49) [139]. A meta-analysis of four RCTs and 11 observational studies showed even greater mortality reduction (OR = 0.12, *p* < 0.001) [140]. These results were confirmed in a critically ill group of mechanically ventilated patients (mortality reduction HR = 0.54; *p* = 0.03) [141]. In addition, an RCT of imatinib, a tyrosine kinase inhibitor that attenuates endothelial vascular leak, was shown to reduce 28-day and 90-day COVID-19 mortality and improve ventilation [142,143]. A clinical trial is underway to determine whether this improvement is due to attenuation of endothelial dysfunction [143].

Severe COVID-19 is often accompanied by severe coagulopathy, venous thrombosis and occasional pulmonary embolization [144,145]. Several RCTs and observational studies have sought to determine the role of anticoagulant treatment in improving patient survival [144,145]. Some RCTs have shown that therapeutic anticoagulation reduces patient mortality [146,147], while others have been stopped for reasons of futility [148]. Observational studies have shown mixed results: some show therapeutic anticoagulation improves mortality [149] while others do not. In ICU patients, an intermediate dose of low molecular weight heparin offers no increase in benefits over a standard dose [150]. Anticoagulation of COVID-19 inpatients with venous thrombosis is recommended but prophylactic anticoagulation of all inpatients is not. Long-term outpatient anticoagulation of discharged patients is not protective. The use of direct acting oral anticoagulants is not recommended; they have not been shown to be effective in reducing mortality. In patients who have experienced venous thrombosis, the duration of anticoagulant treatment after hospital discharge is uncertain.

## 7. Treating the Host Response with Inexpensive Generic Drugs

Severe COVID-19 is associated with dysregulated energy metabolism [40]. Several observational studies have shown that metformin, which acts through AMP-activated protein kinase (AMPK) and PGC-1α to increase mitochondrial biogenesis and improve energy metabolism, reduces COVID-19 mortality in outpatients with Type 2 diabetes mellitus [151]. An RCT of outpatient metformin treatment, however, failed to show this benefit [137].

Fenofibrate is a peroxisome proliferator activated receptor alpha (PPAR-α) agonist that may help minimize the inflammatory and thrombotic consequences associated with SARSCoV-2 infection [152]. It attenuates the interaction between SARS-CoV-2 and ACE2, which could directly reduce infection-related inflammation. Unfortunately, an RCT has shown that fenofibrate has no effect on COVID-19 outcomes [153]. Pioglitazone and rosiglitazone are peroxisome proliferator activated receptor gamma (PPAR-γ,) agonists (thiazolidinediones) that also have anti-inflammatory activities in COVID-19 patients and have been suggested for treatment [154,155]. They too have been shown not to affect outcomes in COVID-19 patients.

## 8. Treating the Host Response to COVID-19 with Inexpensive Generic Statins, ACE Inhibitors (ACEis), and Angiotensin Receptor Blockers (ARBs)

Soon after the onset of the COVID-19 pandemic, an observational study from China reported that inpatient statin treatment was associated with a reduction in mortality [156]. This was thought to be due to the pleiotropic effects of statin treatment on the host response.

I have reviewed the putative mechanisms for these statin effects many times [17,19,22]. and will not repeat them here. Many observational studies have reported that outpatient statins reduce COVID-19 hospitalizations and mortality [157]. These findings also apply to patients with coagulopathies and immunothrombosis [158] and risk conditions such as diabetes [159].

Observational studies have also shown that chronic treatment with ACE inhibitors or ARBs is not harmful and can be beneficial in COVID-19 patients [160,161]. A clinical trial of telmisartan (an ARB) has yielded similar results [162]. Moreover, continuation of ACE inhibitor/ARB outpatient treatment after hospitalization has beneficial effects on COVID-19 outcomes [163], whereas discontinuing treatment can be harmful [164,165,166,167,168]. Similarly, an increase in COVID-19 mortality has been observed following withdrawal of statin treatment [169,170]. Withdrawal of treatment with these drugs is discussed in greater detail below.

Although the complexities of COVID-19 have been extensively reviewed, there is no single consensus on its pathophysiology. Nonetheless, many of the biomarkers associated with COVID-19 hyperinflammation, endothelial dysfunction and immunothrombosis are beneficially affected by both statins and ACEis/ARBs. The effects of these drugs on several important COVID-19 biomarkers are shown in Table 3.

Cardiologists have known for many years that combination treatment with a statin and an ACE inhibitor are synergistic [171,172]. In 2014/2015, a statin/ARB combination appeared to dramatically reduce Ebola mortality in Sierra Leone [21,22,173]. Combination treatment is also discussed below.

For people who live in LMICs, COVID-19 treatments licensed in wealthy countries may be too expensive or they are simply unavailable. Hospital beds for those with critical illness may be few or unavailable [174]. Moreover, statin use itself might be limited [175]. (There are no data on the extent to which ACE inhibitors/ARBs or other generic drugs are used in LMICs.) Nonetheless, in resource-poor countries inexpensive generic drugs like statins, ACE inhibitors, and ARBs might be the only practical way to save the lives of patients with severe COVID-19 [176]. These drugs will be available in all countries on the first pandemic day. In addition. because they target the host response to infection, they could be used to save lives during any pandemic [16,17,18,19,22]. They might even be used to save the lives of those with other forms of acute critical illness like sepsis and ARDS [177].

## 9. Statin and ACE Inhibitor/ARB Withdrawal

Investigators have known for many years that statin treatment is associated with reduced mortality due to several infectious diseases [178]. Cardiovascular investigators know that statins are clearly beneficial in preventing cardiovascular diseases in people less than 75 years in age [179]. Moreover, research published 15–20 years ago showed that statin treatment reduced morbidity and mortality in patients with acute myocardial infarction [180]. It made no difference whether outpatient statins were continued after hospital admission or were started in the hospital. Furthermore, statin withdrawal was associated with an increase in cardiovascular mortality. The importance of statin withdrawal and its probable mechanisms of action were extensively reviewed in 2006 [181]. For COVID-19, similar findings have been published for ACE inhibitor and ARB withdrawal [164,165,166,167,168].

Most studies showing that statins are associated with reduced COVID-19 mortality are based on outpatient treatment [27]. The point estimates for mortality reduction in these studies (e.g., Ref. [157]) are unreliable because they do not document whether statin treatment was continued or withdrawn after hospital admission. Thus, accurate estimates for mortality reduction can only be obtained from evidence of inpatient statin treatment.

## 10. Inpatient Statin Treatment

In 2021, an observational study by Belgian investigators reported that inpatient statin treatment was associated with reduced COVID-19 mortality [26]. At least 24 observational studies have reported similar results [26,158,169,182,183,184,185,186,187,188,189,190,191,192,193,194,195,196,197,198]. The results of six RCTs of inpatient statin treatment have also been reported [199,200,201,202,203,204]. All of these results are summarized in Table 4.

A meta-analysis of five of the six RCTs of inpatient statin treatment has been published [206]. The relative risk of death was 0.90 (95% CI = 0.73–1.11; *p* value = 0.33). Daily statin treatment had no effect on mortality, but three of the six studies were very small [199,200,204], four used what was probably too low a dose of atorvastatin (20 mg instead of 40 mg) [199,200,201,202], and one dealt only with ICU patients [202], which may have been too late in the course of illness. Two RCTs examined simultaneous treatment with several drugs [200,203]. One additional RCT reported the results of statin/aspirin treatment on mortality [207]. In this study, ten days of atorvastatin treatment (40 mg) reduced in-hospital mortality, but no statistical results were reported. An earlier meta-analysis of “inpatient” statin treatment included eight RCTs [208]. Unfortunately, one of the eight observational studies of statin effects on mortality in this meta-analysis reported on chronic (i.e., outpatient) but not inpatient statin treatment.

Of the 22 observational studies of inpatient statin treatment, 12 used propensity score matching to minimize confounding [26,158,182,185,187,189,191,193,194,196,197,205], five reported treating >1000 patients [170,182,184,189,197], and many of the remaining studies included hundreds of subjects. All but one observational study [194] showed that statins significantly reduced COVID-19 mortality. Almost all observational studies recommended that they be followed by RCTs.

Thus, as shown in Table 4, there was a distinct difference in the results of RCTs and observational studies of inpatient statin treatment. Almost all of the observational studies showed mortality reductions, whereas all of the RCTs failed to show these reductions. The REMAP-CAP investigators should soon report findings from a large RCT of inpatient statin treatment. It is hoped these widely anticipated results will help resolve this difference.

## 11. Combination Treatment

In 2021, Belgian investigators published an observational study showing that inpatient treatment with a combination of a statin and either an ACE inhibitor or an ARB was associated with a threefold reduction in 28-day COVID-19 mortality [26]. Combination treatment was more effective in reducing COVID-19 mortality than statin treatment alone.

Combination treatment for pandemics was suggested in 2008 [17]. For COVID-19, combinations of repurposed drugs have been very effective [209]. Earlier studies had shown that a statin/ACE inhibitor combination was effective in reducing morbidity after coronary artery bypass surgery [171]. A statin/ARB combination appeared effective in reducing mortality during the Ebola outbreak in Sierra Leone [21,22,173]. Combination treatment is not unusual; combinations of antivirals and dexamethasone had been reported earlier [210]. Combinations of baricitinib with remdesivir [211] and with dexamethasone [212] have been reported for COVID-19.

The Belgian study is the only report of inpatient combination statin and ACEi or ARB treatment of COVID-19, although this combination has probably been widely used by clinicians (e.g., Ref. [169]). The success of combination polypill treatment for the prevention of cardiovascular disease [21,213,214] suggests the potential for using an inexpensive generic drug combination for treating patients affected by any pandemic. A pragmatic combination could contain two or more generic drugs (a statin, an ACEi/ARB, metformin, or a PPARα or PPARγ agonist). Each of these individual drugs is known to be safe in patients with critical illness and in those requiring long-term treatment. A combination polypill-like treatment could be especially important for pandemic-affected patients in LMICs.

## 12. Why Have There Been No RCTs of Combination Treatment with Statins and ACEis/ARBs?

Other than the Belgian hospital study [26], it is surprising that no studies of combination treatment with statins and ACE inhibitor/ARB treatment have been undertaken. Other combination studies for COVID-19 have been reported [211,212]. One can only speculate about the reasons for the absence of these combination studies.

No pharmaceutical company would make money from advocating generic drug treatment for COVID-19 (although the health benefits for people in LMICs could be substantial [18,25]). Leadership for the global response to the COVID-19 pandemic, especially the rapid development of effective vaccines, has come from WHO, international institutions and prestigious national health agencies. Many of those who have favored vaccines have not been elected by the people in high-income countries who have benefitted most [215]. Nonetheless, global estimates of COVID-19′s excess mortality (at least 20 million excess deaths [1]) strongly suggest that the success of vaccine development has come too late for most of the world’s people.

Inexpensive generic drugs like statins affect many aspects of the host response to infection [216,217]. Three RCTs published more than a decade ago showed that statins alone were ineffective in improving survival in patients with sepsis and ARDS, especially in those admitted to ICUs [218]. These results may have persuaded some health officials and investigators that it would be useless to test statins against COVID-19. In addition, failure to understand the importance of subphenotypes in determining responses to statin treatment may have led many to conclude that statins were ineffective [219]. Moreover, social influences and behavioral biases may have led some to overlook or dismiss the idea that statins might be helpful [220,221]. The advice of scientific experts about COVID-19 was generally accepted (despite uncertainties) by most people. Support for political leaders was initially high, but distrust soon arose because political decisions often differed from the views of scientists [222]. This lack of trust led to difficulties with vaccination programs and might have led some to conclude that host response treatments had nothing to offer.

## 13. The Way Forward—The Next Pandemic

The COVID-19 pandemic might be on the decline, but the SARS-CoV-2 virus is not going to go away [223]. Most virologists predict it will become endemic. A few regions of the world might be able to eliminate the virus but only if herd immunity levels (induced by vaccination or previous infection) are very high. No one can predict how the virus will evolve, but its evolution is certain [224]. Future ‘variant waves” might be characterized by increased mortality or be benign like those of other coronaviruses that humans have experienced for several decades. Whether affordable and effective treatments will eventually be discovered and widely implemented to blunt these waves is uncertain [225]. In the meantime, the burden of the current pandemic must not be forgotten—more than 20 million excess deaths worldwide [1] and more than an estimated 10 million orphans [226]. This burden has fallen heavily on LMICs [227].

*The Lancet* recently published the findings and recommendations of its COVID-19 Commission [228]. The findings were predictable and unremarkable—the pandemic’s origin remains unknown, the reactions of WHO and national governments were too slow, public opposition to advice indicated a lack of trust, widespread inequities occurred everywhere (especially access to rapidly developed vaccines in LMICs). Social and economic progress were set back in all countries. Many of the Commission’s recommendations are self-evident—strengthening national health systems, expanding national pandemic preparedness planning and improving “mass vaccination, availability and affordability of testing, treatment for new infections and long COVID (test and treat), complementary public health and social measures (including the wearing of face masks in some contexts), promotion of safe workplaces, and economic and social support for self-isolation” [228]. However, other recommendations such as establishing a WHO Science Council, a World Health Assembly-sponsored Global Health Board and a new WHO-based Global Health Fund, if implemented, are unlikely to make a difference when the next pandemic arrives.

An agenda for pandemic research by clinicians is shown in Table 5 [16,17,18,19,22,28,229]. It is unlikely that arguments regarding the primacy of RCTs over observational studies will be settled anytime soon, although the concept of “real world evidence” (RWE) and the availability of electronic health records (EHRs) have allowed the utility of observational studies (i.e., most RWE studies) to be more widely discussed [69,230,231,232,233,234,235,236,237,238,239,240]. In contrast, critics of RWE have written about why they favor RCTs over observational studies [241,242]. Those who favor RWE studies say that having to choose between the two is a ‘myth’. Although neither method is perfect, the two are synergistic and they complement each other [233]. While “early observational studies and small randomized trials may cause spurious claims of effectiveness”, this conclusion is based on an examination of antiviral agents, not host response treatments [243]. Sometimes, clinicians are justified in undertaking innovative treatment before conducting definitive research [244] as long as it is supported by other RWE data [238]. The goal of all research “must be actionable data—data that are sufficient for clinical and public health—that have been derived openly and objectively and that enable us to say, “’Here’s what we recommend and why’” [245]. As I wrote more than ten years ago, “Sadly, the arithmetic for pandemic vaccines and antivirals is unforgiving. WHO is focused on vaccines and antivirals that will only be available to people who can afford them, and that’s ten percent of the world’s population. Consequently, it doesn’t matter that arguments for their use are scientifically well grounded; in practical terms they are pointless, in the same way that it is pointless to tell a starving man he should eat if there’s no food in the kitchen. For pandemic vaccines and antiviral agents, the kitchen is empty. We should stop talking about things that people in developing countries will never have, and start talking about things they’ve already got” [246].

Research on acute and long COVID-19 [247,248] will continue. In addition to pathophysiological studies (Figure 1) related to endothelial dysfunction, innate and adaptive immunity, interferon and abnormal coagulation, observational studies will continue to examine host response treatments [249,250], especially target trial studies that emulate RCTs [127]. The unexplained “tolerance” of children compared with adults will still require explanation [23,251,252,253]. In addition, energy metabolism, epigenetic changes, and the contributions of the microbiome and circadian rhythms to COVID-19 pathogenesis will come to the fore. New treatments and treatment combinations will receive more attention. Moreover, clinical and epidemiological studies will begin to document outpatient drug treatments that might influence COVID-19 hospitalizations and outcomes as much as high-risk conditions and abnormal laboratory findings.

Research for pandemic preparedness must consider the needs of people who live in LMICs, recognizing that every life-saving discovery will also help those who live in rich countries. As discussed in this review, treating pandemic patients with generic drugs like statins and ACEis/ARBs could “nudge” the host response back toward self-regulating homeostasis. It might not have much effect on the infection itself, but it might save lives. There is no guarantee it would work, but good science demands it be tried. A journalist has recently written about the challenge we face. “We should see science as something people do: as a way of solving problems, a project that does not just describe the world but brashly wants to change it. A science that people will follow must lead. If in the next pandemic we want something else from our public health leaders—to save lives and not tear the country apart in the process—we must learn to see science as a vehicle, not a dodge, for human agency: something we are right to make demands of, right at times to get angry at, whose terrible failures it must own along with its triumphs” [254].

## Figures and Tables

**Table 1:** Strengths and limitations of RCTs and observational studies.

	RCTs	Observational Studies
Strengths	Randomization balances baseline characteristics	Rigor is enhanced by specific methods
	“Prospective” infrastructure collects pertinent data	Observational studies and RCTs with the same focus provide consistent results
	Analytic methods are simple and straightforward	Treatments evaluated in large populations can be shown to be safe and effective
Limitations	Individual RCTs are often contradictory	Baseline characteristics are usually not well balanced
	Meta-analyses and large trials often disagree	Data quality can be variable
	Limited generalizability	Analytical methods can be complex and obscure

Adapted from Ref. [67].

**Table 2:** Summary of guidelines for the use of treatments for acute COVID-19.

Treatment Guidelines	NIH Guidelines	ERS Guidelines
**Antiviral Treatments Targeting the SARS-CoV-2 Virus**		
HCQ/CQ	Not recommended	Strongly not recommended
Remdesivir	Recommended	Conditionally recommended
Favipiravir	Not mentioned	Not mentioned
Convalescent plasma	Not recommended	Not recommended
Ivermectin	Not recommended	Strongly not recommended
Interferon–1β	Not mentioned	Conditionally not recommended
Pegylated interferon-lambda	Not mentioned	Not mentioned
Monoclonal antibodies specific for the anti-SARS-CoV-2 spike protein	Not mentioned	Recommended
Paxlovid (Ritonavir-boosted nirmatrelvir	Recommended	Not mentioned
Molnupiravir	Weakly recommended	Not mentioned
Colchicine	Not recommended	Strongly not recommended
**Immunomodulators targeting the host response to infection**		
Corticosteroids	Recommended, requiring O_2_ treatment only	Strongly recommended
mAb—IL-1 receptor antagonist	Not recommended *	Conditionally not recommended
mAb—IL-6 receptor antagonist	Recommended	Strongly recommended
Fluvoxamine (SSRI)	Not recommended *	Not mentioned
Janus kinase inhibitors	Strongly recommended	Strongly recommended
Tyrosine kinase inhibitors	Not recommended *	Not mentioned
Anticoagulation (LMWH)	Recommended	Strongly recommended
Azithromycin	Not recommended	Not mentioned
Azithromycin + HCQ	Not recommended	Not mentioned
**Inexpensive generic drugs targeting the host response**		
Metformin	Not recommended *	Not mentioned
PPARα, PPARγ agonists	Not mentioned	Not mentioned
Statins, ACE inhibitors, ARBs	Not mentioned except for continuing treatment	Not mentioned

Adapted from Refs. [77,78,79]. Abbreviations: NIH = National Institutes of Health; ERS = European Respiratory Society; HCQ = hydroxychloroquine; CQ = chloroquine; mAb = monoclonal antibody; Paxlovid = ritonavir-boosted nirmatrelvir; SSRI = selective serotonin uptake inhibitor; Not recommended * = Not recommended except when used in a clinical trial; LMWH = low molecular weight heparin; PPAR = peroxisome proliferator activator receptor; ACE = angiotensin converting enzyme; ARB = angiotensin receptor blocker.

**Table 3:** Beneficial effects of statin, ACE inhibitor or ARB treatment on biomarkers of inflammation and endothelial barrier integrity.

Biomarker	Improve Inflammation/ Endothelial Barrier Integrity			Biomarker	Improve Inflammation/ Endothelial Barrier Integrity			Biomarker	Improve Inflammation/ Endothelial Barrier Integrity		
	Statin	ACEi	ARB		Statin	ACEi	ARB		Statin	ACEi	ARB
Tyrosine kinase	yes	yes	yes	PAF	yes	yes	yes	PPAR α	yes	yes	yes
Janus kinase	yes	yes	yes	PAR-1/PAR-2	yes	yes	yes	PPAR γ	yes	yes	yes
IL-1	yes	yes	yes					α7 nicotinic aCh receptor	yes	yes	yes
IL-4	yes	yes	yes	ROS	yes	yes	yes	RAGE	yes	yes	yes
IL-6	yes	yes	yes	β-arrestin	yes	yes	yes	Ferritin	yes	yes	yes
IL-10	yes	yes	yes	Inflammasome	yes	yes	yes	Mitochondria	yes	yes	yes
IL-17	yes	yes	yes	AMPK	yes	yes	yes	HO-1	yes	yes	yes
TNF	yes	yes	yes	MAPK/Akt	yes	yes	yes	KLF4	yes	yes	yes
HMBG1	yes	yes	yes	MCP-1	yes	yes	yes				
Lipoxin A4	yes	yes	yes	FOXP3	yes	yes	yes	Angpt2/ Tie2	yes	yes	yes
				T regs	yes	yes	yes	ACE2	yes	yes	yes
HMBG1	yes	yes	yes	NADPH oxidase	yes	yes	yes	eNOS/ iNOS	yes	yes	yes
Thrombomodulin	yes	yes	yes	Interferon	yes	yes	yes	VCAM-1/ ICAM-1	yes	yes	yes
Thromboxane A2	yes	yes	yes	TGF-β1	yes	yes	yes	VE- cadherin	yes	yes	yes
t-PA	yes	yes	yes	hs CRP	yes	yes	yes	Actin cytoskeleton	yes	yes	yes
P-selectin/ E-selectin	yes	yes	yes	mTOR	yes	yes	yes	VEGF	yes	yes	yes
PAI-1	yes	yes	yes	Adiponectin	yes	yes	yes	Bradykinin	yes	yes	yes

Adapted from Ref. [22] and updated. The biomarkers shown in this table are representative and do not include all that affect inflammation or endothelial barrier integrity. Inflammatory biomarkers and endothelial barrier disruptors and protectors are signaling molecules or pathways. Beneficial treatment by statins, ACE inhibitors (ACEis) or angiotensin receptor blockers (ARBs) is defined as either up regulation or down regulation in cell signaling pathways that reduce inflammation and/or improve endothelial barrier integrity. This is indicated by “yes in the table. The literature for each agent on each biomarker in Table 3 is extensive and individual articles have not been cited. Three documents showing selected citations and abstracts for these articles are available from the author (davidsfedson@gmail.com). Abbreviations: α7 nicotinic aCh receptor = alpha7 nicotinic acetylcholinesterase receptor; ACE2 = angiotensin converting enzyme-2; Angpt = angiopoietin; AMPK = adenosine monophosphate kinase; C = complement; eNOS/iNOS = endothelial/inducible nitric oxide synthase; FOXP3 = fork head box P3; HMGB1 = high mobility group box 1; hsCRP = highly sensitive C-reactive protein; HO-1 = heme oxygenase-1; IL-1 = interleukin 1; IL-4 = interleukin 4; IL-6 = interleukin 6; IL-10 = interleukin 10; IL-17 = interleukin 17; KLF4 = Kruppel-like factor 4; MAPK/Akt = mitogen-activated protein kinase/three members of the serine/threonine protein kinase family; MCP-1 = monocyte chemoattractant protein-1; MMPs = matrix metalloproteinases; mTOR = mechanistic target of rapamycin kinase; PAF = platelet activating factor; PAI-1 = plasminogen activator inhibitor-1; PAR = protease activator receptor; PPARα = peroxisome proliferative activated receptor alpha; PPARγ = peroxisome proliferative activated receptor gamma; P-selectin = platelet selectin; E-selectin = endothelial selectin; RAGE = receptor for advanced glycation end products; ROS = reactive oxygen species; TGF-β1 = transforming growth factor-β1; tPA = tissue plasminogen activator; Treg = regulatory T cells; TNF = tumor necrosis factor; VCAM-1/ICAM-1 = vascular/intercellular adhesion molecule-1; VE-cadherin = vascular endothelial cadherin; VEGF = vascular endothelial growth factor.

**Table 4:** Twenty-two observational studies and six RCTs of inpatient statin treatment and its effectiveness in reducing 28–30-day mortality.

Study (Ref.)	Methods	No. of Statin Users	Adjusted OR/HR	95% CI	*p* Value
Zhang [182]	PSM (4:1), CCS	1219	0.58	0.43–0.80	0.001
Rodriguez-Nava [183]	ICU only, cohort, Cox regression	ns	0.38	0.18–0.77	0.008
Mallow [184]	Cohort, multivariate regression	5313	0.54	0.49–0.60	<0.001
Saeed [185]	Diabetes mellitus, multivariate regression	982	0.51	0.43–0.61	<0.001
	PSM (1:1), IPTW *, diabetes vs. no DM,		0.88	0.84–0.91	<0.001
Lala [186]	Adjusted for HRC, ACEi/ARB	984	0.57	0.47–0.69	<0.001
Fan [187]	PSM (1:1), cohort	250	0.25	0.07–0.92	0.037
Rossi [188]	Observational study, compares only lipophilic/hydrophilic statins; no adjustment for HRC or other risk variables	42	ns	-	0.025
Torres-Pena [189]	PSM (1:1), statins continued vs. withdrawal **, mixed effect logistic regression	1130	0.67	0.54–0.84	<0.001
Byttebier [26]	PSM (1:1), CCS	297	0.56	0.39–0.93	0.020
Terleki [190]	Logistic regression	ns	0.54	0.33–0.84	0.008
Lohia [191]	PSM (1:1), cohort	250	0.47	0.32–0.70	<0.001
Choi [192]	Cox regression, high intensity statin	843	0.53	0.43–0.65	not done
Davoodi [199]	RCT, atorvastatin, 20 mg for 5 days	20	no deaths	-	-
Shen [193]	PSM (1:1), logistic regression	404	0.47	0.29–0.77	<0.001
Ayeh [194]	PSM (1:1), Cox regression	594	0.92	0.53–1.59	ns
Masana [195]	GM (1:1)	336	0.60	0.39–0.92	0.020
Memel [169]	marginal structural Cox regression, IPTW, statin treatment vs. no treatment	777	0.57	0.37–0.86	0.008
	statins continued vs. withdrawal ***	-	0.27	0.11–0.64	0.003
Matli [200]	RCT, Cox regression, atorvastatin 20 mg + other drugs	17	1.43	0.28–13.16	0.644
Ghafoori [201]	RCT, Cox regression, atorvastatin 20 mg	76	ns (multiple outcomes including ICU admissions and deaths	ns	0.27
I.S.Investigators [202]	RCT, ICU, atorvastatin 20 mg	210	0.84	0.58–1.22	0.39
Gaitan-Duarte [203]	RCT, rosuvastatin 40 mg + other drugs	159	0.53	0.29–0.56	0.038
Kuno [196]	PSM (1:1), statins continued vs. withdrawal	671	0.53	0.41–0.62	<0.001
Li [197]	PSM (1:1)	3359	0.72	0.64–0.80	<0.001
Kouhpeikar [198]	Cox regression, composite outcome (mortality, ICU, ventilation)	162	0.57	0.33–0.99	0.048
Andrews [170]	Logistic regression	26,893	0.72	0.68–0.77	<0.001
Al Harbi [158]	PSM (1:1), ICU, Cox proportional hazard regression	198	0.72	0.54–0.97	0.030
Al-Sulaiman [205]	PSM (1:1), ICU, Cox proportional hazard regression	251	0.75	0.58–0.98	0.03
Hejazi [204]	RCT	26	ns (mortality was twice as high in control patients)	ns	ns

Adapted from Ref. [27] and updated. Abbreviations: CCS = case-control study; CI = confidence interval; GM = genetics-matched; HR = hazard ratio; HRC = high risk conditions; ICU = intensive care unit; IPTW = inverse probability treatment weighted; ns = not stated or not significant; OR = odds ratio; PSM = propensity score-matched; RCT = randomized controlled trial. * The PS matched IPTW cohort analysis included demographic and comorbidity factors, clinical and laboratory test values, and the use of ACE inhibitors and angiotensin receptor blockers. ** Statin treatment continued after hospital admission versus statin withdrawal; conditional logistic regression. *** Statin treatment continued after hospital admission versus statin withdrawal; marginal structural Cox model.

**Table 5:** A research agenda for clinicians in treating the host response to COVID-19 and other pandemic illnesses.

*Choose Drugs That Are*
Known to modify the host response to infection; Safe in patients with acute critical illness; Inexpensive generics; Widely available in low- and middle-income countries; Familiar to practicing physicians; Likely to affect meaningful outcomes (such as 28–30-day mortality).
*Plan Clinical studies of Host Response Treatment*
Consult with investigators who understand the biology of the host response (e.g., vascular biology, mitochondrial biogenesis, disease tolerance, immunometabolism); Study inexpensive generic drugs as monotherapy or in combinations; Undertake observational studies (using target trials methods) in patients hospitalized with COVID-19; Undertake prospective clinical trials in patients hospitalized with COVID-19; Undertake the same studies in patients hospitalized with everyday acute critical illnesses, including seasonal influenza, community-acquired pneumonia, sepsis; Study outcomes in children and adults; Evaluate outcomes (e.g., 28–30-day mortality) following individual and combination drug treatment.
*Plan What to Do with the Results*
Identify local sources of supply for potentially efficacious generic drugs; Determine quantities usually supplied and capacities for surge production; Assess patterns of distribution, needs for stockpiling, and logistics for delivery; Determine drug costs for public programs; Prepare to communicate study results to physicians, health officials, and the public.

Adapted from Ref. [25] and updated.

## Data Availability

Not applicable.
